# Association Between Osteoporosis and Refracture Rate Among Patients With Hip Fractures at King Abdulaziz Medical City, Saudi Arabia

**DOI:** 10.7759/cureus.22171

**Published:** 2022-02-13

**Authors:** Ali H AlYami, Majed N Alosaimi, Mohammed S Alshehri, Abdulhamid T Alghamdi, Majd A SaemAldahar, Turki A Alsafrani, Albaraa A Dabroom, Ibrahim A Kattan, Fares M Sindi, Azzam M Azaya, Bandar N AlMaeen

**Affiliations:** 1 Department of Surgery, Ministry of National Guard Health Affairs, Jeddah, SAU; 2 Department of Surgery, King Saud Bin Abdulaziz University for Health Sciences, Jeddah, SAU; 3 Department of Orthopaedic Surgery, Ministry of National Guard Health Affairs, Jeddah, SAU; 4 Department of Orthopaedics, King Abdullah International Medical Research Center, Riyadh, SAU; 5 Department of Orthopaedics, King Saud Bin Abdulaziz University for Health Sciences, Jeddah, SAU; 6 College of Medicine, Ministry of National Guard Health Affairs, Jeddah, SAU; 7 College of Medicine, King Saud Bin Abdulaziz University for Health Sciences, Jeddah, SAU; 8 Department of Surgery, College of Medicine, Jouf University, Al-Jouf, SAU; 9 Department of Orthopaedic Surgery, King Faisal Specialist Hospital and Research Centre, Riyadh, SAU

**Keywords:** fracture, hip, dexa, refracture, osteoporosis

## Abstract

Background

Hip fracture is a major medical and surgical topic and is a significant cause of morbidity and mortality. Older women, especially those with osteoporosis, are at an increased risk for hip fractures. Multiple studies have shown the effect of osteoporosis on the refracture rate among the elderly population. Therefore, selecting a targeted population for screening and treating osteoporosis has an essential role in decreasing the hip fracture rate. This study aimed to determine the association between osteoporosis treatment and refracture rate among patients with hip fractures at King Abdulaziz Medical City, Jeddah, Saudi Arabia.

Methods

Collected data included patient demographics (men: ≥55 years old; women: ≥50 years old), the used osteoporosis investigation method, osteoporosis treatment history, presence of comorbidities, and refracture as a primary outcome. The refracture rate among patients with hip fracture was calculated and used to determine the association between hip refracture and osteoporosis.

Results

Our study included a total of 292 patients who presented to our hospital due to hip fractures. The patients were divided into two groups, the osteoporotic and non-osteoporotic groups. These groups were then compared. There was no statistical significance between osteoporosis and hip refracture (p = 0.721), and there was no association between the treatment of osteoporosis and hip refracture (p = 0.493). Statistical difference was found between patients who had undergone dual-energy X-ray absorptiometry scan and were not treated for osteoporosis (p = 0.00). Lastly, the mortality of the refracture group was 10%, while it was 11% in the no-refracture group (p = 1.00).

Conclusion

Morbidity and mortality rates are higher among patients with hip fractures. Our study showed that there was no association between hip refracture rate and osteoporosis whether the patient is treated for osteoporosis or not. We recommend a systematic review that can include more studies in this field to acquire more definitive results regarding this topic.

## Introduction

Hip fracture is a major health problem, especially in the elderly population, and is a significant cause of mortality and morbidity. The incidence of hip fracture in the United States is 80 per 100,000, and the incidence doubles each decade in patients over 50 years old. The prevalence of hip fracture is two to three times higher in women than men [1]. The mortality rate following hip fracture was reported to range between 14% and 37% within 12 months in different studies [2-4]. Different risk factors are related to hip fracture, such as decreased bone mineral density (BMD), decreased visual acuity, neuromuscular impairment, and cognitive impairment [5].

Osteoporosis is a skeletal disorder characterized by reduced bone strength leading to an increased risk for fracture. In this disorder, bone quality is compromised, bone strength is weakened, and most importantly, patients with osteoporosis are at an increased risk for fracture and refracture. Several studies worldwide demonstrated that there is a higher rate of refracture following an osteoporotic fracture, and the rate is approximately two to nine times higher among the elderly population. As shown in different studies worldwide, failure to adhere to therapy was the leading cause of refracture [6,7]. Osteoporosis-related fractures may have potentially catastrophic consequences, including disability and death [8]. Osteoporosis affects approximately 200 million people worldwide [9]. Screening for osteoporosis is ideally initiated in all women aged ≥65 years old by measuring BMD of the hip and lumbar spine using dual-energy X-ray absorptiometry (DEXA).

Despite the current recommendation suggested by the United States Preventive Services Task Force (USPSTF) in osteoporosis screening, recent studies show that only 25% of women aged 65-85 are screened for osteoporosis [10]. Furthermore, DEXA scan results are inadequately followed in treating patients undergoing osteoporosis screening. For example, one study showed that 33% of patients who underwent DEXA did not receive treatment for abnormal results, and most did not have their results reviewed [11].

Multiple studies in different countries with variable results reported the importance of treating osteoporosis and its association with reducing hip refracture rate and mortality [12,13,14]. However, local data on this topic are lacking. Thus, this study aimed to determine the association between osteoporosis and refracture rate among patients with hip fractures at King Abdulaziz Medical City, Jeddah, Saudi Arabia.

## Materials and methods

This retrospective cohort study included all patients diagnosed with hip fracture, fractures in the femoral head, neck of femur, and intertrochanteric and per-trochanteric fractures. Male patients ≥55 years old and female patients ≥50 years old were included in this study at King Abdulaziz Medical City in Jeddah, Saudi Arabia. Data were gathered using a data collection sheet to collect patient demographics, such as age, gender, weight, and height, the used osteoporosis investigation method, osteoporosis treatment history, the presence of co-morbidities, and the outcome. All data were acquired from Best Care System using electronic medical records at King Abdulaziz Medical City. The refracture rate among patients with hip fracture was calculated and used to determine the association between hip refracture and osteoporosis as a primary objective. A DEXA scan was used as the method for screening and diagnosing osteoporosis. Primary treatments for osteoporosis included denosumab, teriparatide, and alendronate. Calcium and vitamin D supplements were not considered as primary treatments for osteoporosis. Our primary outcome was hip refracture, including the type of refracture and the occurrence of death from refracture.

The sample size was calculated using ClinCalc.com by setting the lifetime incidence of hip fracture to 15-18%. Moreover, the anticipated fracture rate in our study is 30%, with a margin of error of 5%, and the power of the study is 80%. The total sample size was 630, with 315 in each group. However, a non-probability consecutive sampling technique was used to include all patients who met the inclusion and exclusion criteria. Data were collected by the co-authors of the study. Ethical approval was obtained from the institutional review board, and scientific approval was obtained from King Abdullah International Medical Research Center.

IBM SPSS version 23 was used for data analysis. Categorical variables were reported as percentages, and numerical variables were reported as means or medians. Normally distributed numerical variables were reported using the mean and SD, and skewed distributed variables were reported using median and interquartile range (IQR). Chi-squared test and Fisher’s exact test were used to analyze the association between osteoporosis and refracture rate, and it was also used in other variables, such as the DEXA scan results, to analyze their relation to patients treated and not treated for osteoporosis. Statistical significance was set at a p-value of <0.05.

## Results

We conducted a retrospective cohort study that included a total of 292 patients who have been subjected to hip fracture in our hospital. The median age in the osteoporotic group is 74 years, while in the non-osteoporotic group is 73 years. Most patients were females in the osteoporotic group (66.7%) and males in the non-osteoporotic group (58.5%). The most common fracture was femoral neck fracture (50% in females and 49.5% in males). Diabetes mellitus and hypertension were prevalent in both groups (50.7% and 47.9% in females; 72% and 58.5% in males, respectively). Other demographics and co-morbidities are summarized in Table 1.

**Table 1:** General demographics. *: Chi-squared test; **: Fisher’s exact test; IQR: Interquartile range.

Demographics		Osteoporotic	Non-osteoporotic	p-value
Sex	Male	25 (33.3%)	127 (58.5%)	0.00*
	Female	50 (66.7%)	90 (41.5%)	
Calcium/Vitamin D supplements	Yes	55 (73.3%)	78 (35.9%)	0.00*
	No	20 (26.7%)	139 (64.1%)	
Type of fracture of the first fracture	Acetabular fracture	1 (1.4%)	9 (4.2%)	0.71*
	Femoral neck fracture	37 (50%)	106 (49.5%)	
	Intertrochanteric Fracture	35 (47.3%)	96 (44.9%)	
	Subtrochanteric fracture	1 (1.4%)	3 (1.4%)	
	Total fractures	74 (100%)	214 (100%)	
Diabetes mellitus	Yes	38 (50.7%)	104 (47.9%)	0.68*
	No	37 (49.3%)	113 (52.1%)	
Hypertension	Yes	54 (72%)	127 (58.5%)	0.038*
	No	21 (28%)	90 (41.5)	
Dyslipidemia	Yes	19 (25.3%)	36 (16.6%)	0.095*
	No	56 (74.7%)	181 (83.4%)	
Congestive heart failure	Yes	12 (16%)	19 (8.8%)	0.079*
	No	63 (84%)	198 (91.2%)	
Arthritis	Yes	16 (21.3%)	15 (6.9%)	0.00*
	No	59 (78.7%)	202 (93.1%)	
Chronic kidney disease	Yes	15 (20%)	35 (16.1%)	0.44*
	No	60 (80%)	182 (83.9%)	
Hypothyroidism	Yes	6 (8%)	12 (5.5%)	0.41**
	No	69 (92%)	205 (94.5%)	
Hyperparathyroidism	Yes	1 (1.3%)	2 (0.9%)	1.00**
	No	74 (98.7%)	215 (99.1%)	
Cancer/Tumor	Yes	8 (10.7%)	25 (11.5%)	0.84*
	No	67 (89.3%)	192 (88.5%)	
Age, Median (IQR)		74 (18)	73 (25)	
BMI, Median (IQR)		26.6 (8.4)	25.4 (7.5)	

Table 2 demonstrates the primary objective outcome comparing the refracture in both groups with osteoporosis. Most patients in the refracture and no-refracture groups were non-osteoporotic (70% and 74.5%, respectively). No statistical significance was found using Fisher’s exact test between both groups (p = 0.721). We compared the refracture rate with treatment effect to further assess the impact of the treatment. Most patients in the refracture and no-refracture groups did not receive treatment. Fisher’s exact test showed no statistical significance between the refracture group and osteoporosis treatment (p = 0.493).

**Table 2:** Primary objective outcome comparing the refracture in both groups with osteoporosis. **: Fisher’s exact test.

Outcome		Refracture	No refracture	p-value
Osteoporosis	Yes	3 (30%)	72 (25.5%)	0.721**
	No	7 (70%)	210 (74.5%)	
Treated osteoporosis (in osteoporotic group)	Yes	1 (33.3%)	14 (19.4%)	0.493**
	No	2 (66.7%)	58 (80.6%)	

To assess the investigation across the sample, we compared the patients who underwent a DEXA scan and started to receive treatment with those who did not. Seventy-eight patients had done the scan, yet only 14 out of those 78 received treatment. Statistical significance was found between the groups (p = 0.00). The distribution of both groups is shown in Table 3.

**Table 3:** Investigation outcome. **: Fisher’s exact test.

Factor		Treated for osteoporosis	Not treated for osteoporosis	p-value
DEXA scan	Yes	14 (17.9%)	64 (82.1%)	0.00**
	No	3 (1.4%)	211 (98.6%)	

The mortality of the refracture group is 10% (1/10), while that of the no-refracture group is 11%. Table 4 shows no statistical significance between mortality and refracture (p = 1.00).

**Table 4:** Mortality in the refracture groups. **: Fisher’s exact test.

Outcome		Died	Did not die	p-value
Refracture	Yes	1 (10%)	9 (90%)	1.00**
	No	31 (11%)	251 (89%)	

## Discussion

Our analysis showed that in patients with a history of a hip fracture, 26.71% were screened for osteoporosis using a DEXA scan. This percentage is comparable to the numbers reported in similar studies. According to Murray AW et al., the population in their study involved two centers. Patients who were offered or already had undergone a DEXA scan were 19.4% in one center and 40.5% in another center [15]. Similarly, in an interventional study by Davis JC et al., 29% of patients had received a DEXA scan after a hip fracture. This number was significantly increased compared to their control population [16].

Since populations who suffer from osteoporosis are usually elderly, they have a higher possibility of having other co-morbidities; therefore, it is essential to discuss the possible association of osteoporosis with different factors. For instance, there was no significant association between type 2 diabetes mellitus and osteoporosis in our study. Saller A et al. reviewed multiple articles regarding this association, which reported contradicting findings; some studies reported a higher BMD in patients who had diabetes, while other studies noticed sex-related differences in BMD changes [17]. Data remain unclear regarding the association between these diseases. However, it is essential to consider other factors that contribute to fractures. For example, visual impairment and peripheral neuropathy associated with type 2 diabetes may precipitate falls, increasing the risk of fractures.

Our study reported a significant statistical association between arthritis and osteoporosis. Patients with osteoporosis were less likely to have arthritis; however, studies by Geusens PP et al. and Bultink IE et al. have reported their coexistence. They were considered to coexist due to factors related to body composition and the underlying inflammatory processes. In addition, there are other similar demographic factors, such as age and gender [18,19].

Of the 75 patients diagnosed with osteoporosis, only 20% were treated for osteoporosis. This number is low compared to the study by Satomi et al., in which 43% of the patients with previously diagnosed osteoporosis have received treatment [19]. This difference is mainly attributed to the definition of the treatment of osteoporosis. In the study by Satomi E et al., supplementation with calcium only was considered a treatment; however, it was not in our study. Let's consider these modalities to be a proper treatment. The percentage of treated osteoporotic patients will increase to 41.35%, comparable to multiple other studies, such as those by Satomi E et al. and Smith MD et al. [20, 21].

In measuring the outcome of patients, we investigated the incidence of refracture and mortality rate. Although patients with a history of hip fracture who are osteoporotic are expected to have a higher rate of refracture, our results did not show this. Only 4.44% of the osteoporotic group experienced a refracture. In contrast, the study by Bliuc D et al., which only included osteoporotic patients, reported a refracture rate of 34.59% [22]. Furthermore, Ryg J et al. showed that the hip refracture rate was 11-23% [23]. However, this discrepancy may be because only a quarter of our study population had osteoporotic screening and other fracture risk assessments after their initial fracture. In addition, most studies investigating refractures also included non-hip fractures, while our study excluded them. Moreover, longer follow-up periods may predispose patients to a higher number of refracture incidences, which was included in the study by Ryg J et al. [23]. Misclassification of other complications, such as infections, periprosthetic fractures, and hip refractures, may overestimate and exaggerate the actual percentages. The other measured outcome was the mortality rate. Based on our patient population, the mortality rate was 10.95%, with no significant difference between individuals with and without a refracture. Keene GS et al. reported a 5-year mortality rate of 56%, with some sex-related differences [24]. Also, Bliuc D et al. reported a mortality rate attributed to hip fracture compared to populations of similar age, the female sex in 27%, and the male sex in 24% [22]. The lower refracture rate could explain our lower mortality rate.

Limitation

The sample size was one of the limitations of our study, which affected finding a statistical significance for our objectives. Another limitation is collecting data from a single center. In addition, considering other risk factors, such as obesity, mobilizing status, and other chronic diseases, may add value to our results. Finally, considering other sites of refracture, such as in the ribs and vertebral fractures, would change the refracture rate in our study.

## Conclusions

Hip fracture is a significant health issue and is an important cause of morbidity and mortality, especially in the elderly population. Our study suggests that there was no association between the refracture rate and osteoporosis, regardless of whether the patient was treated for osteoporosis or not. However, we recommend utilizing a DEXA scan to detect osteoporosis in patients and manage it to improve the outcome of the fracture rate. Moreover, we recommend a systematic review that can include more studies in this field to acquire more definitive results regarding this topic.
